# Association between 25(OH)D Level, Ultraviolet Exposure, Geographical Location, and Inflammatory Bowel Disease Activity: A Systematic Review and Meta-Analysis

**DOI:** 10.1371/journal.pone.0132036

**Published:** 2015-07-14

**Authors:** Chao Lu, Jun Yang, Weilai Yu, Dejian Li, Zun Xiang, Yiming Lin, Chaohui Yu

**Affiliations:** Department of Gastroenterology, the First Affiliated Hospital, College of Medicine, Zhejiang University, Hangzhou, 310003, China; University Hospital Llandough, UNITED KINGDOM

## Abstract

**Background:**

There is no consensus on the vitamin D levels and inflammatory bowel disease (IBD).

**Aim:**

To conduct a systematic review and meta-analysis to analyze the relationship between IBD and 25(OH)D, sun exposure, and latitude, and to determine whether vitamin D deficiency affects the severity of IBD.

**Methods:**

We searched the PubMed, EBSCO, and ClinicalTrials.gov databases to identify all studies that assessed the association between 25(OH)D, sun exposure, latitude, and IBD through November 1, 2014, without language restrictions. Studies that compared 25(OH)D levels between IBD patients and controls were selected for inclusion in the meta-analysis. We calculated pooled standardized mean differences (SMDs) and odds ratios (ORs).

**Results:**

Thirteen case-control studies investigating CD and 25(OH)D levels were included, and eight studies part of above studies also investigated the relationship between UC and 25(OH)D. Both CD patients (SMD: 0.26 nmol/L, 95% confidence interval [CI]: 0.09–0.42 nmol/L) and UC patients (SMD: 0.5 nmol/L, 95% CI: 0.15–0.85 nmol/L) had lower levels of 25(OH)D than controls. In addition, CD patients and UC patients were 1.95 times (OR, 1.95; 95% CI, 1.48–2.57) and 2.02 times (OR, 2.02; 95% CI, 1.13–3.60) more likely to be 25(OH)D deficient than controls. We also included 10 studies investigating the relationship between CD activity and vitamin D. Results showed that patients with active CD (CD Activity Index≥150) were more likely to have low vitamin D levels. In addition, whether low sun exposure and high latitude were related to a high morbidity of CD need to be provided more evidence.

**Conclusion:**

Our study shows that IBD patients have lower vitamin D levels. For active CD patients, vitamin D levels were low. These findings suggest that vitamin D may play an important role in the development of IBD, although a direct association could not be determined in our study.

## Introduction

Inflammatory bowel disease (IBD) including Crohn's disease (CD) and ulcerative colitis (UC), refers to a group of chronic gastrointestinal disorders characterized by dysregulated intestinal inflammation[1]. IBD can be caused by environmental factors[2], a genetic susceptibility such as NOD2 mutation[3] or interleukin (IL)-23 receptor mutation[4], immune dysregulation[5], or other factors. As a public health problem, IBD has a high incidence in developed and northern countries, and although still low, the incidence of IBD in developing areas is increasing[2, 6].

Some studies have suggested that vitamin D as a immunoregulatory element plays an important role in the occurrence and progression of IBD[7, 8]. Vitamin D is an indispensable element in the human body that is found in two main forms: storage-type 25(OH)D, which is hydroxylated by the liver, and active-type 1,25(OH)_2_D, which is hydroxylated by the kidney. It is also known as a “sunshine element”, because it can be synthesized upon exposure to ultraviolet (UV) rays. The biological effects of vitamin D are primarily mediated by the vitamin D nuclear receptor (VDR)[9], and it is widely known to act in the metabolism of bone. However, the discovery of VDR expression in more target tissues, such as the kidney, thyroid, intestine, skin, immune cells, nonparenchymal hepatocytes, and biliary epithelial cells[10, 11], suggests that vitamin D may influence systemic human metabolism including the immune system. Mouli et al identified an association between vitamin D deficiency and the development of IBD and also found that vitamin D deficiency influences disease severity[12]. The immunomodulatory function of vitamin D and immune factors in the pathogenesis of IBD suggest that vitamin D levels are commonly low among patients of IBD. In addition, people living in areas at high latitude that receive little sun expose synthesize low levels of vitamin D and have higher incidence. Such observations provide supporting evidence for the relationship between vitamin D and IBD.

In this study, we conducted a meta-analysis and systematic review to analyze the relationships between IBD and vitamin D, sun exposure, and latitude. We also aimed to determine whether vitamin D deficiency directly affects the severity of IBD.

## Methods

### Data sources and study selection

We searched articles published from January 1, 2000 to November 1, 2014 in PubMed, ClinicalTrials.gov, and EBSCO databases without language restrictions. Following terms: *inflammatory bowel disease*, *IBD*, *Crohn's disease*, *CD*, *ulcerative colitis*, *UC*, *vitamin D*, *25(OH) vitamin D*, *25(OH)D* were used. “OR” was used as the set operator to combine different sets of results. We selected studies that reported data on vitamin D levels, sun exposure, latitude, and IBD with or without mention of disease severity. Controlled clinical trials investigating vitamin D levels and IBD were included in order to conduct a meta-analysis so that we can conclude whether vitamin D is related to IBD. Then, a systematic review was conducted instead of meta-analysis regarding the relationship between vitamin D and IBD activity due to the small number of high quality papers and lacking of data. In addition, we examined correlations between vitamin D and latitude or annual sunshine exposure in the cities where the included studies were performed in order to assess whether living in areas at high latitude or with low sunshine exposure was related to IBD. Age, sex, and other confounding factors were also considered. We excluded papers that did not provide original data, animal studies, in vitro studies, and studies focused on conditions affected by vitamin D metabolism.

### Data abstraction

We abstracted main study characteristics, including patient/volunteer characteristics (including sex, age, and number of people), research country, publication year, serum vitamin D levels as continuous variable, and the cut-off level used to define vitamin D deficiency as dichotomous variable. We obtained data for latitude and annual sunshine exposure according to the cities where the included studies were conducted. Data for annual sunshine exposure were obtained from the Web of the Hong Kong Observatory (http://gb.weather.gov.hk/contentc.htm). All data were double checked by one author. Two investigators independently examined and selected papers for inclusion in our analyses. Another two investigators assessed the quality of papers by applying the Strengthening the Reporting of Observational studies in Epidemiology (STROBE) checklist[13] or other tools[14, 15].

### Statistical analysis

We combined the standardized mean difference (SMD) for studies that reported mean and standard deviation (SD) values for vitamin D levels of IBD patients and controls. 25(OH)D was the main form of vitamin D considered in the included studies. The cut-off level for 25(OH)D deficiency was defined as less than 50 nmol/L[16]. For two studies[17, 18] that provided 95% confidence intervals (CIs) instead of SDs, we obtained a final SD using a reduction formula[19]. Because of the different characteristics between CD and UC, we analyzed them independently. To correct for bias in a small sample size, we used a random effects model. In our study, three studies involving pediatric participants were included[18, 20, 21]. Considering the possible differences in vitamin D levels between children and adults, we also opted to stratify our analyses in pediatric and adult participants. For studies that reported vitamin D deficiency as a dichotomous variable, we pooled the ORs using the Mantel-Haenszel method with a fixed effects model. In addition, the linear regression analysis was used to correlate vitamin D and latitude, annual sunshine exposure. Statistical heterogeneity was assessed by Cochran’s Q-test and the I^2^ statistic. We assessed publication bias with Egger’s test. All analyses were carried out through the application of the commands metan and metabias in STATA 12. Linear regression analysis was operated in SPSS 17.0 (IBM, Chicago, IL, USA).

## Results

### Basic characteristics

**Fig 1:**
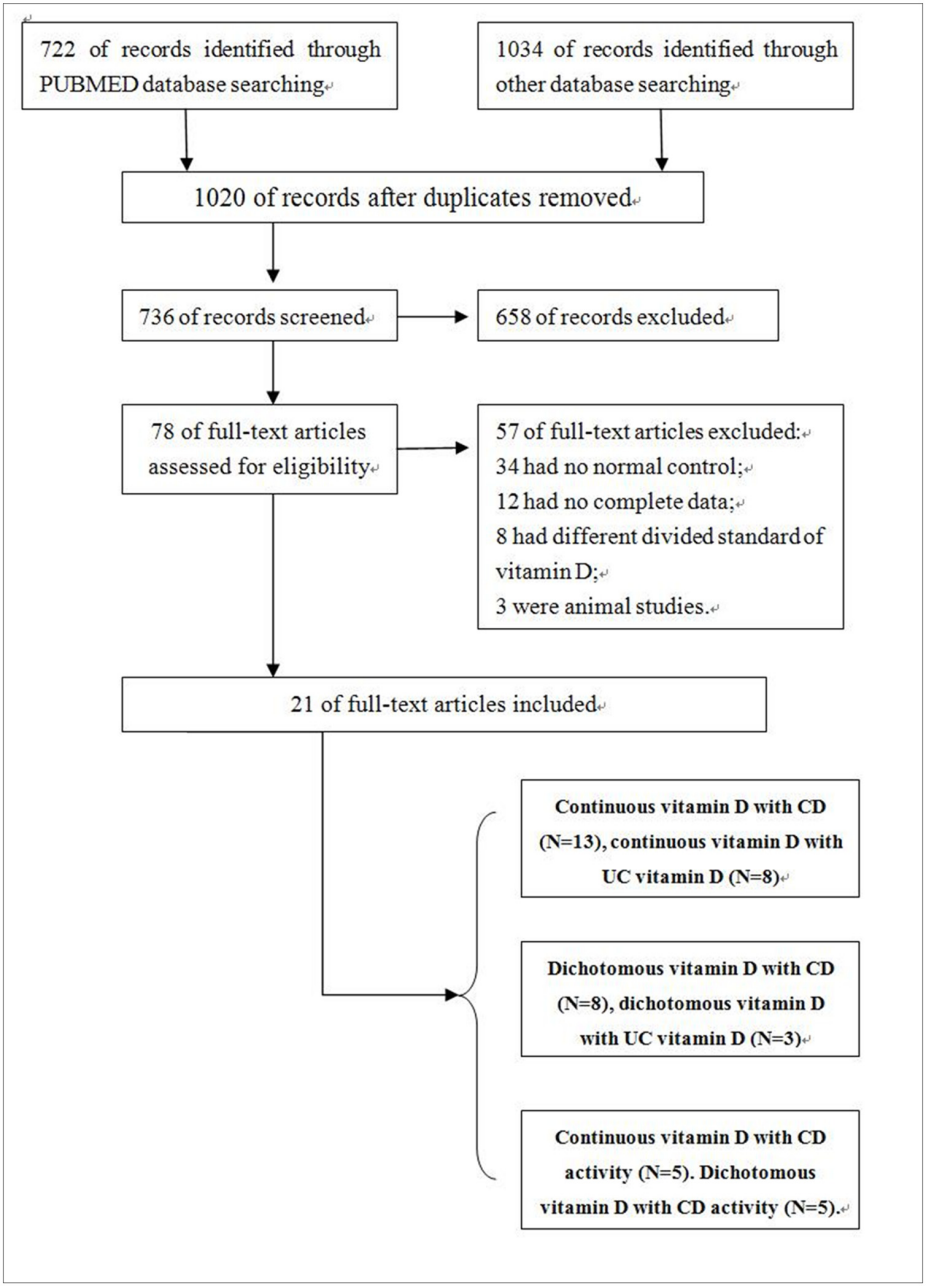
Flow diagram of searching.

Our search identified 1756 related references, of which 21 papers met our inclusion criteria (Fig 1). The reasons for exclusion were shown in Fig 1. The 21 studies included 5 studies conducted in Europe, 9 in North America, 4 in Asia, 1 in Brazil, and 2 in Australia. Our study included 13 studies investigating the association between CD and vitamin D levels [17, 18, 20–30]. Of them, eight studies also referred to the relationship between UC and vitamin D levels[17, 20, 21, 23, 24, 27, 29, 30]. Patients’ basic characteristics are presented in Table 1. However, disease durations, years of follow-up since diagnosis, and surgical treatment were not included due to the lack of data. The participants in three studies[18, 20, 21] were children, and those in the other studies were adults. In addition, eight studies were used to analyze dichotomous exposure (vitamin D deficiency) in CD[21–24, 26, 28, 31, 32], of which three studies also referred to UC[21, 23, 24]. Continuous variable can directly reflect the relationship between IBD and vitamin D levels, whereas dichotomous variables indicate morbidity of IBD under different vitamin D classifications. The combination of continuous and dichotomous variables can reflect the relationship between IBD and vitamin D better. Among 21 studies included, we also analyzed 10 studies to investigate the relation of vitamin D and IBD activity[23, 25, 29, 30, 33–38].

**Table 1:** Studies on vitamin D levels in CD patients, UC patients, and controls.

Author year (ref)	CD(n)/ UC(n)/ Control(n)	Assay method for vitamin D	Age (CD/UC/Control)	Gender (male/female) (CD;UC;Control)	25(OH)D in CD(nmol/L) (mean±SD)*	25(OH)D in UC (nmol/L) (mean±SD)*	25(OH)D in Controls(nmol/L) (mean±SD)*	*P* #
**Joseph 2009 [26]**	**34/0/34**	**Radioimmunoassay**	**39.2 ± 12.9/-/ 38.9 ±13.4**	**24/10;-; 24/10**	**40.6±27**	**-**	**56.9±29.7**	**<0.05**
**Jørgensen 2013 [25]**	**182/0/62**	**Chromatography**	**36 (17–78)/-/32 (18–62)** **	**78/104;-; 30/32**	**69±33**	**-**	**65±25**	**NS** ***
**Garg 2013 [17]**	**40/31/23**	**Electrochemilumine-scence assay**	**41 (23–76)/41 (23–76)/39 (22–68)** **	**22/18;17/14;10/13**	**70±36.3**	**70±32.7**	**66±43.5**	**NS/NS**
**El-Matary 2011 [20]**	**39/21/56**	**Competitive protein binding assay**	**12.2±3.2/12.4±37/ 11.3±4.2**	**19/20;10/11;25/31**	**66.7±27.3**	**56.9±22**	**81.7±15.4**	**<0.05/<0.05**
**Grunbaum 2013 [24]**	**34/21/48**	**Radioimmunoassay**	**39.9 ± 12.3/44.2 ± 13.7/39.6 ± 13.8**	**13/21;8/13;10/38**	**71.1±31.1**	**71.4±36.3**	**68.3±26.2**	**NS/NS**
**Tajika 2004 [29]**	**33/11/15**	**Competitive protein binding assay**	**37.6±7.5/47.6±12.4/37.7±10.0**	**25/8;6/5;8/7**	**37.9±16.2**	**43.9±11.7**	**42.2±13**	**<0.05/<0.05**
**Dumitrescu 2014 [23]**	**14/33/94**	**Chromatography**	**36 ± 9/42 ± 14/42 ± 12**	**8/6;17/16;50/44**	**57.4±25**	**59.9±27.5**	**77.4±32.4**	**<0.05/<0.05**
**Veit 2014 [21]**	**40/18/116**	**Chemiluminescent immunoassay**	**16.61±2.20/16.13±1.99/14.56±4.35**	**24/16;7/11;49/67**	**61.7±24.4**	**53.3±25.5**	**65.3±28**	**NS/NS**
**de Bruyn 2014 [22]**	**101/0/41**	**Chemiluminescent immunoassay**	**41 (30–50)/-/28 (24–39)** **	**31/70;-;8/33**	**51.6±26.6**	**-**	**60.8±27.6**	**NS**
**Suibhne 2012 [28]**	**81/0/70**	**Radioimmunoassay**	**36.43±11.00/-/36.34±9.53**	**32/49;-;28/42**	**47.6±27.2**	**-**	**51.9±24.5**	**NS**
**Middleton 2013 [18]**	**52/0/40**	**Competitive protein binding assay**	**17.0(15.0–18.5)/-/ 11.0(5.0–15.0)** **	**32/20;-;15/25**	**40.2±15.7**	**-**	**40.7±16.2**	**NS**
**Souza 2008 [27]**	**39/37/40**	**-**	**32.1±8.7/35.0±8.5/34.0±7.0**	**21/18;12/25;16/24**	**64.6±20.5**	**54.4±20**	**85.9±31.9**	**<0.05/<0.05**
**Tan 2014 [30]**	**107/124/122**	**ELISA**	**38.0 ± 15.3/39.6 ± 14.4/39.43 ± 12.71**	**68/39;63/61;67/55**	**28.9±12.5**	**25.8±11.1**	**32.1±11**	**<0.05/<0.05**

*Vitamin D levels reported as nmol/L. Vitamin D levels measured in ng/mL were converted to nmol/L by multiplying by 2.496.

** Median and range

***NS, not significant

^#^ *P* compares CD patients or UC patients to controls.

### Association of IBD with vitamin D

**Fig 2:**
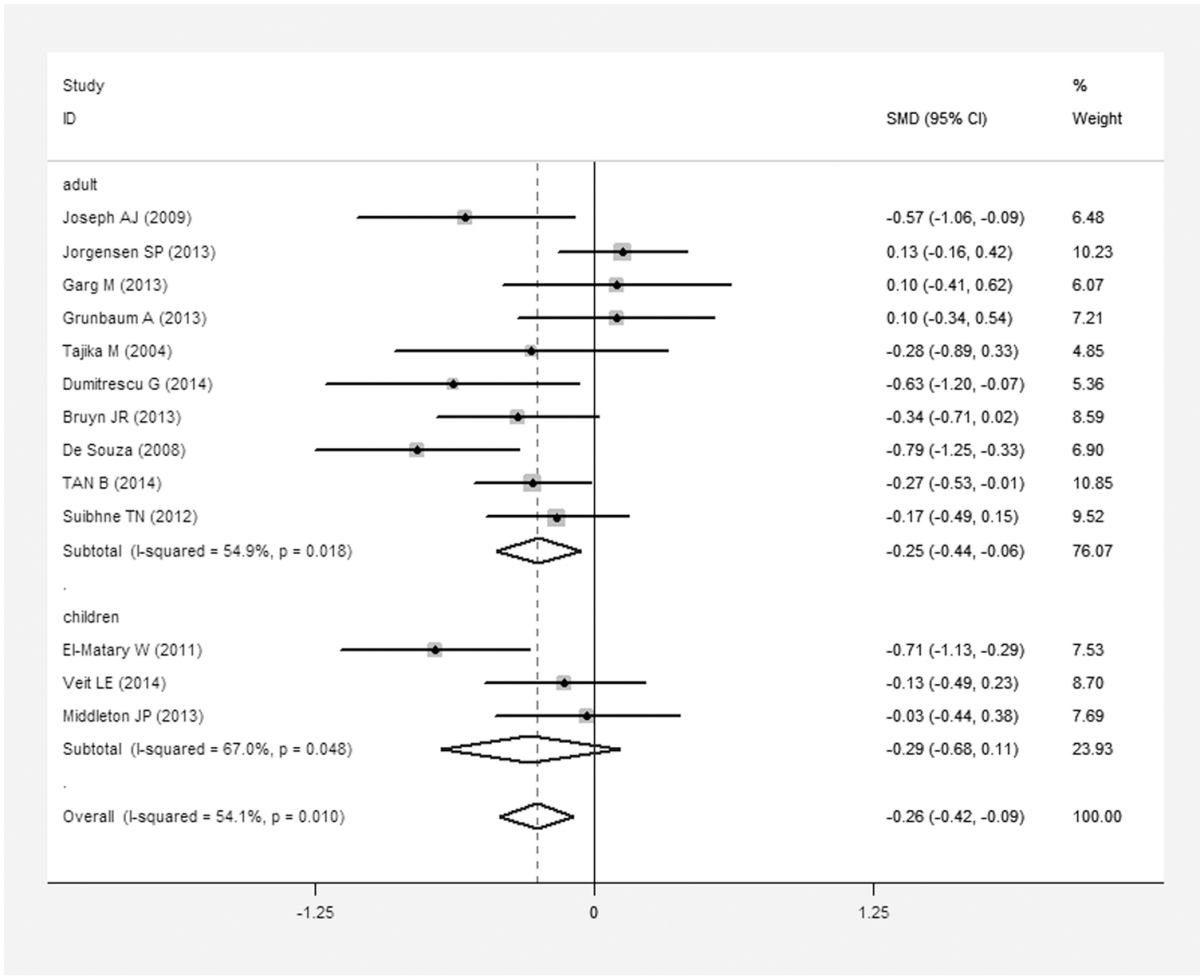
Meta-analysis of studies reporting 25(OH)D levels in CD patients vs. controls, a standardized mean difference with a 95% confidence interval and weight percentage. Subtotals of adults and children, and overall population.

We conducted a meta-analysis of vitamin D levels and CD based on data for 796 CD patients and 761 controls. The average 25(OH)D level in CD patients was 0.26 nmol/L less than that in controls (SMD = 0.26 nmol/L, 95% CI = 0.09–0.42 nmol/L, I^2^ = 54.1%, *P* = 0.01; Fig 2). When we excluded children (131/796 CD patients, 212/761 controls), we found that the average 25(OH)D level in adult CD patients (665/796) was 0.25 nmol/L lower than that in controls (549/761) (SMD = 0.25 nmol/L, 95% CI = 0.06–0.44 nmol/L, I^2^ = 54.9%, *P* = 0.018). In contrast, no statistically significant difference was found between the 25(OH)D levels of pediatric CD patients and controls.

**Fig 3:**
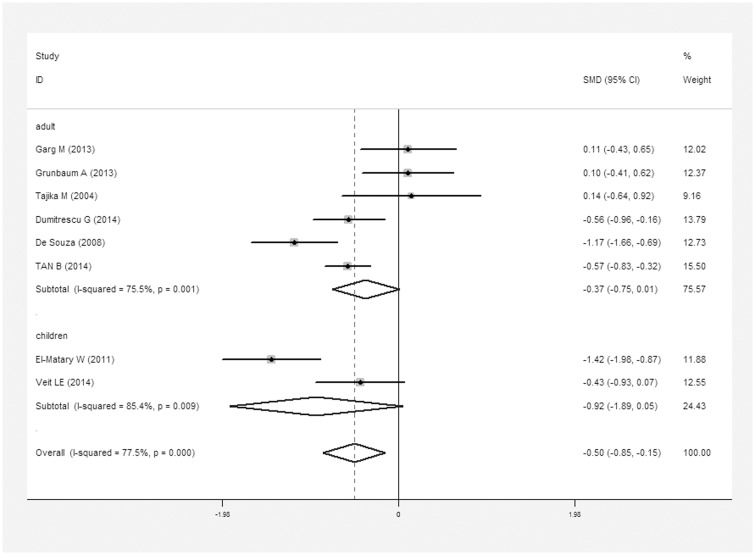
Meta-analysis of studies reporting 25(OH)D levels in UC patients vs. controls, a standardized mean difference with a 95% confidence interval and weight percentage. Subtotals of adults and children, and overall population.

For the meta-analysis of vitamin D levels and UC, 296 UC patients and 514 controls were included. On average, the average 25(OH)D level in UC patients was 0.5 nmol/L lower than that in controls (SMD = 0.5 nmol/L, 95% CI = 0.15–0.85 nmol/L, I^2^ = 77.5%, *P*<0.01; Fig 3). However, neither adult UC patients (218/257) nor pediatric UC patients (39/257) had significantly different levels from controls independently. Different methods for quantifying vitamin D levels did not show changes in the inferences.

**Fig 4:**
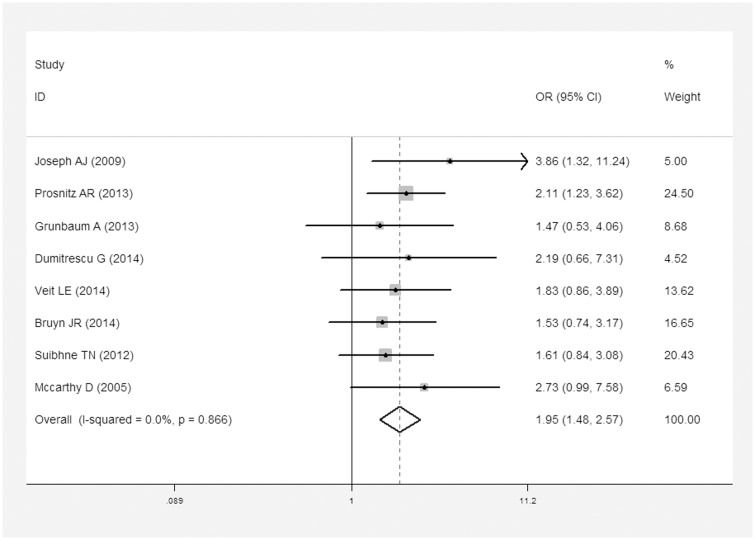
Meta-analysis of studies reporting dichotomous outcomes of 25(OH)D levels in CD patients vs. controls and estimated OR with a 95% confidence interval and weight percentage.

**Fig 5:**
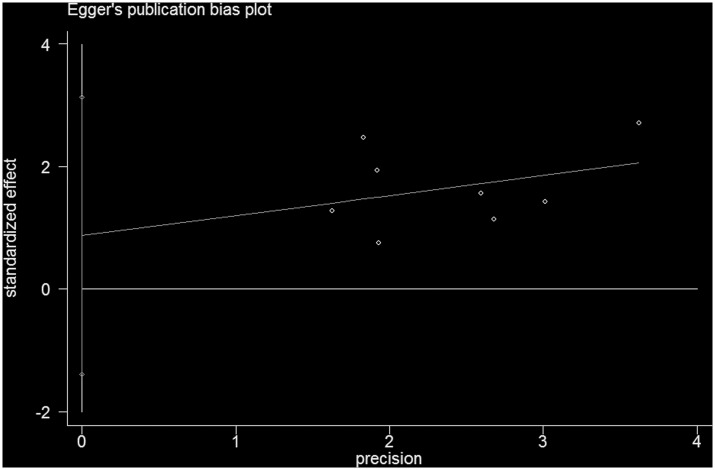
Egger’s test results for publication bias about dichotomous outcomes of 25(OH)D levels in CD.

**Fig 6:**
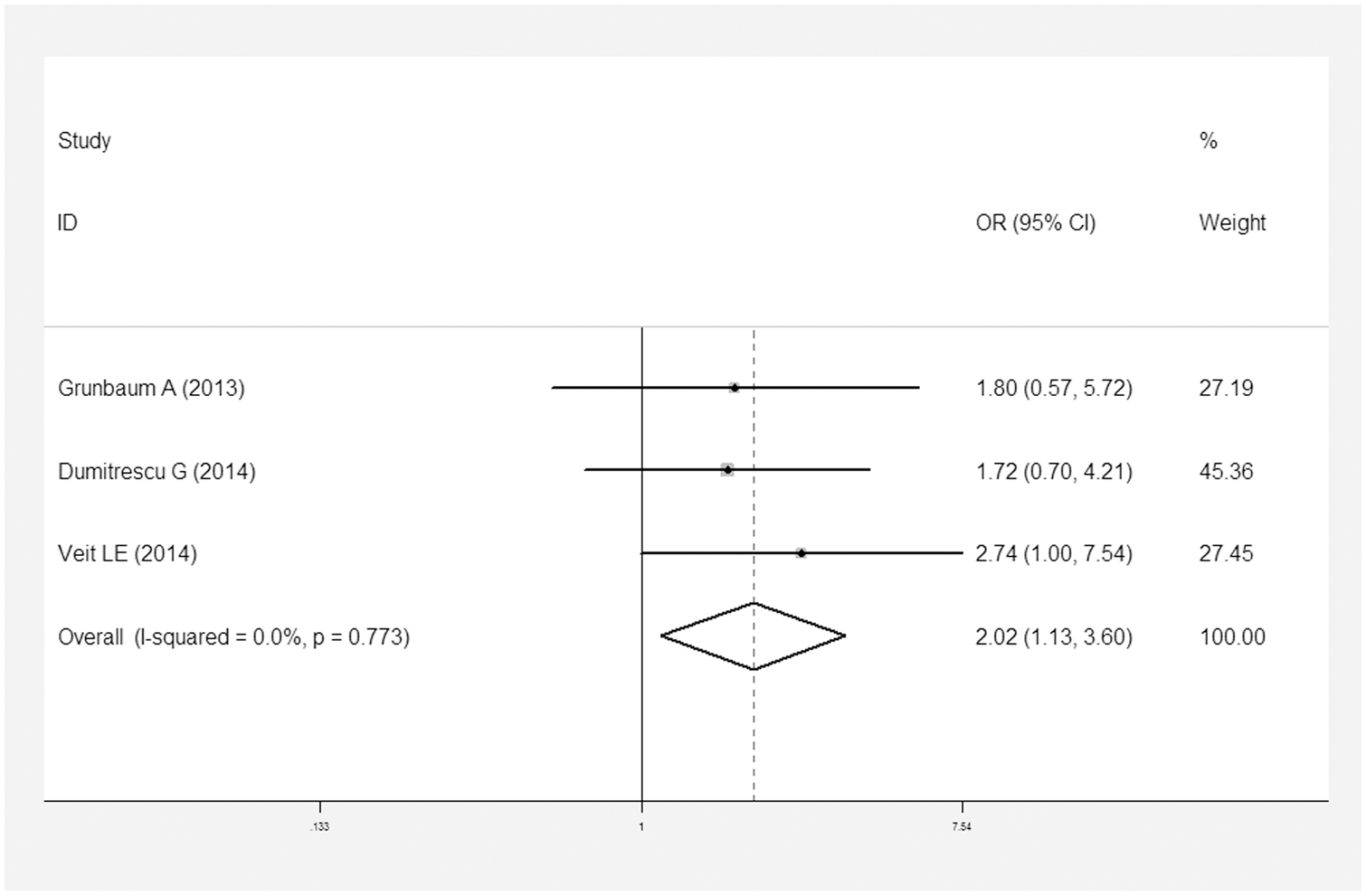
Meta-analysis of studies reporting dichotomous outcomes of 25(OH)D levels in UC patients vs. controls and estimated OR with a 95% confidence interval and weight percentage.

In addition, eight reports described dichotomous variables for vitamin D deficiency in patients with CD [21–24, 26, 28, 31, 32]. Three studies investigated UC and vitamin D deficiency [21, 23, 24](Table 2). CD patients were 1.95 times (OR = 1.95, 95% CI = 1.48–2.57, I^2^ = 0, *P* = 0.866) more likely to suffer vitamin D deficiency than controls (Fig 4). And no publication bias was found under Egger’s test (*P* = 0.383, Fig 5). Similarly, UC patients were 2.02 times more likely to experience vitamin D deficiency than controls (OR = 2.02, 95% CI = 1.13–3.60, I^2^ = 0, *P* = 0.773; Fig 6).

**Table 2:** Data for vitamin D deficiency among CD patients, UC patients, and controls.

Author/year (ref)	CD(n)/ UC(n)/ Control(n)	Age (CD/UC/ Control)	Gender (male/female) (CD;UC;Control)	CD Deficiency (n)	CD Sufficiency (n)	UC Deficiency (n)	UC Sufficiency (n)	Control Deficiency (n)	Control Sufficiency (n)	*P* *
**Joseph 2009 [26]**	**34/0/34**	**39.2 ± 12.9/-/38.9±13.4**	**24/10;-;24/10**	**27**	**7**	**-**	**-**	**17**	**17**	**<0.05**
**Prosnitz 2013 [32]**	**78/0/221**	**12.7±2.8/-/ 13.5±4.4**	**44/34;-;112/109**	**33**	**45**	**-**	**-**	**57**	**164**	**<0.05**
**de Bruyn 2014 [22]**	**101/0/41**	**41 (30–50)/-/28 (24–39)** **	**31/70;-;8/33**	**55**	**46**	**-**	**-**	**18**	**23**	**NS** ^**#**^
**Suibhne 2012 [28]**	**81/0/70**	**36.43±11.00/-/36.34±9.53**	**32/49;-;28/42**	**51**	**30**	**-**	**-**	**36**	**34**	**NS**
**McCarthy 2005 [31]**	**44/0/44**	**36.7±11.0/-/ 36.9±11.1**	**15/29;-;15/29**	**15**	**29**	**-**	**-**	**7**	**37**	**NS**
**Grunbaum 2013[24]** ***	**34/21/48**	**39.9±12.3/ 44.2±13.7/ 39.6±13.8**	**13/21;8/13;10/38**	**10**	**21**	**7**	**12**	**11**	**34**	**NS/NS**
**Dumitrescu 2014 [23]**	**14/33/94**	**36±9/42±14/ 42±12**	**8/6;17/16;50/44**	**5**	**9**	**10**	**23**	**19**	**75**	**NS/NS**
**Veit 2014 [21]**	**40/18/116**	**16.61±2.20/ 16.13±1.99/ 14.56±4.35**	**24/16;7/11;49/67**	**16**	**24**	**9**	**9**	**31**	**85**	**NS/<0.05**

* *P* compares CD or UC patients to controls.

** Median and range

***Because of some missing data on serum 25[OH] vitamin D levels, the number of deficiency and sufficiency together didn't match the total number.

^#^NS, not significant

### Association of IBD activity with vitamin D

Our results supported the conclusion that CD and UC patients have lower vitamin D levels than control individuals. Based on this phenomenon, we hypothesized that vitamin D levels are lower in patients with active IBD. To investigate this hypothesis, we collected only articles regarding CD, because the number of studies in UC patients was too few. First, we assessed disease activity according to the Crohn’s Disease Activity Index (CDAI) as described by Best et al [39] or the Harvey-Bradshaw index (HBI) presented by Harvey et al [40]. A CDAI <150 or HBI score <5 was defined as CD remission, whereas a CDAI ≥150 or HBI score ≥5 was defined as activity. The Pediatric Crohn’s Disease Activity Index (PCDAI) was applied in pediatric cases of CD. Ten studies conformed to the requirements and were divided into a continuous variable group [23, 25, 30, 34, 35] (Table 3) and a dichotomous variable group [29, 33, 36–38] (Table 4). Due to the lack of data, a meta-analysis was not possible. In the continuous variable group, four studies supported our hypothesis that CD patients with a CDAI<150 or HBI<5 had higher vitamin D levels compared to those in patients with active CD. The remaining study [35] reported that vitamin D levels with CDAI<150 was higher, but not significantly (P = 0.389), which didn’t statistically support our hypothesis, possibly because their study population was relatively small. In the dichotomous variable group, only one study [38] indicated that CD patients with vitamin D deficiency had a higher activity score than controls (*P* = 0.002). The other four studies found no statistical difference about active score between vitamin D deficiency and sufficiency (Table 4). This may have been affected by differences in the cut-off value for vitamin D deficiency or the bias of subjects. More data and studies are needed to draw an exact conclusion, but currently, we determined that CD patients in remission were more likely to have higher vitamin D levels than those with active disease.

**Table 3:** Data for continuous variable group on vitamin D and CD activity.

Author year (ref)	N(active/ inactive)	Age	Vitamin D levels (active/inactive)(nmol/L)	Country	Type of CD activity	Results
**Jørgensen 2013 [25]**	**-**	**-**	**-/64** *	**Denmark**	**CDAI** **	**Vitamin D level with CDAI<150 was higher than that for mild or moderate CD activity (*P*<0.01)**
**Hassan 2013 [35]**	**14/12**	**34 ± 18**	**23.2 ± 18.5/29.5 ± 2.0**	**Iran**	**CDAI**	**Vitamin D level with CDAI<150 was higher, but not significantly (*P* = 0.389)**
**Dumitrescu 2014 [23]**	**-**	**36 ± 9**	**-/64.9 ±17.5**	**Romania**	**CDAI**	**CD patients with a CDAI≥150 had significantly lower vitamin D levels than those with a CDAI<150 (*P*<0.05)**
**Ham 2014 [34]**	**20/17**	**-**	**67.4 ± 5.0/94.8 ± 7.5**	**USA**	**HBI** ***	**Vitamin D level in patients with active disease was lower than that in patients in remission (*P* = 0.02)**
**Tan 2014 [30]**	**-**	**-**	**-/32.8 ± 13.6**	**China**	**HBI**	**Patients with active disease had significantly lower levels of vitamin D than those in remission (*P*<0.05)**

* We could not combine data to calculate vitamin D levels of active

**CDAI: Crohn’s Disease Activity Index

***HBI: Harvey-Bradshaw index

**Table 4:** Data for dichotomous variable group on vitamin D deficiency and CD activity.

Author year (ref)	Cut-off of vitamin D deficiency (nmol/L)	N(VD deficiency/without deficiency)	Active score(VD deficiency/without deficiency)	Country	Type of CD activity	Results
**Tajika 2004 [29]**	**25**	**9/24**	**111.8±46.7/ 73.8±39.3**	**Japan**	**CDAI** *	**CDAI was higher in patients with vitamin D deficiency, but not significantly(*P*>0.05)**
**Levin 2011 [36]**	**50**	**-**	**10.5±6.9/ 13.1±11.2**	**Australia**	**PCDAI** **	**No differences between the two groups**
**Ulitsky 2011 [38]**	**50**	**241/263**	**3.55/2.61**	**USA**	**HBI** ***	**HBI in patients with vitamin D deficiency was higher (3.55 vs. 2.61, *P* = 0.002)**
**Sentongo 2002 [37]**	**38**	**18/94**	**17.08±11.17/ 11.63±12.52**	**USA**	**PCDAI**	**No differences between the two groups**
**Fu 2012 [33]**	**50**	**17/23**	**-**	**Canada**	**HBI**	**No differences between the two groups**

*CDAI: Crohn’s Disease Activity Index

**PCDAI: Pediatric Crohn’s Disease Activity Index

***HBI: Harvey-Bradshaw index

### Latitude, annual sunshine exposure, vitamin D and IBD

Economou et al [41] confirmed that the incidence of IBD is higher in countries with low sun exposure and located at a high latitude. We also tried these findings. It is well known that vitamin D synthesis depends on exposure to sunlight and solar ultraviolet radiation, which is affected by latitude, season, and duration of daily sunshine[42]. We correlated vitamin D levels with the latitude and annual sunshine exposure of the cities where the included studies were conducted. Due to a lack of adequate data, we found no correlation between vitamin D and latitude or vitamin D and annual sunshine exposure in CD (r = 0.069, P = 0.856; r = -0.439, P = 0.265, respectively) or in control (r = -0.355, P = 0.344; r = -0.745, P = 0.059, respectively). In addition, three studies [43–45] studying the link between sunshine and IBD showed that low sun exposure was highly associated with CD, but not UC. Limketkai et al also indicated that lower sunshine exposure is associated with greater rates of hospitalization, prolonged hospitalization, and the need for bowel surgery [44]. With respect to latitude and IBD, two large-scale perspective studies from the USA [46] and Europe [47] reported an association between latitude and IBD. As a result of the limitations of included articles, we cannot directly prove that lower sun exposure and higher latitude lead to a higher incidence of IBD. Generally, more studies investigating correlations between vitamin D levels and IBD are needed and then we can merge the data to obtain more reliable results regarding correlations between vitamin D levels and latitude or annual sunshine exposure in future analyses.

## Discussion

From this systematic review and meta-analysis, we concluded that vitamin D levels are strongly associated with IBD. We found lower vitamin D levels in CD and UC patients. Moreover, CD and UC patients were 1.95 times and 2.02 times, respectively, more likely to be vitamin D deficient. We also found that vitamin D levels influence the activity of CD.

In our analysis of vitamin D related to latitude and annual sunshine exposure, we expected a negative correlation between vitamin D and latitude, but a positive correlation between vitamin D and annual sunshine exposure. The relationship with latitude was roughly as expected (the correction in IBD was very low with a positive correlation factor of 0.069), whereas that with sunshine exposure was opposite to our expectation. Confounding factors, such as Human Development Index, working environment (malnutrition low-income populations are more inclined to work outdoor), the vitamin D content of food, and the frequency of outdoor exercise that we could not analyze may have influenced the results. In addition, based on our data, we observed that the trend between vitamin D levels with respect to latitude and sunshine exposure was more obvious in control patients, which is consistent the hypothesis that there may be a relationship between vitamin D levels and IBD activity due to lower vitamin D levels in IBD patients.

Yang et al indicated that maximal vitamin D supplementation with 5,000 IU/d can significantly increased serum vitamin D levels from 16±10 ng/ml to 45±19 ng/ml (*P*<0.0001) and reduce the unadjusted mean CDAI scores by 112±81 points from 230±74 to 118±66 (*P*<0.0001)[48]. In addition, in Bendix-Struve’s study, patients treated with high dose vitamin D (1200 IU/d) showed CDAI scores reduced from 37 (range 8–111) to 34 (range 23–53). Meanwhile, CDAI scores in the control group (placebo treated) increased from 23 (range 0–187) to 45 (range 0–273)[49]. These results suggest that vitamin D supplementation can alleviate the severity of IBD and further provide evidence for the role of vitamin D in the development of IBD.

As an intestinal disease, the influence of gastrointestinal inflammation in the pathogenesis of IBD could not be ignored. Papadakis et al confirmed that CD and UC are related to specific cytokine profiles: interferon (IFN)-γ, tumor necrosis factor (TNF)-α, and interleukin (IL)-12 levels are elevated in CD, whereas IL-5 is increased in UC[50]. These specific pro-inflammatory cytokines were found in the inflamed mucosa of CD and UC patients. Meanwhile, vitamin D plays an important role in the immune system[51]. It can mediate T helper type 1 (Th1) cells, which can produce pro-inflammatory cytokines including IFN-γ, IL-2, and TNF-α [52, 53]. Muller et al indicated that vitamin D can inhibit the release of TNF-α [54], and VDR-/- mice showed more production of TNF-α, IL-6, and IL-1β[55], illustrating the important role of VDR in IBD. Obviously, the immunologic and inflammatory relationships between IBD and vitamin D were responsible for the results of our study. In addition to immune factors, vitamin D signaling can regulate the expression of NOD2 [56] and autophagy homeostasis including TNF-α–induced autophagy[57], which may also play an important role in IBD.

Additional experimental evidence from animal studies further supports the immunologic role of vitamin D and VDR in IBD. VDR-/- mice treated with dextran sodium sulfate (DSS) showed elevated levels of IFN-γ and TNF-α and had significantly fewer intact crypts, more intestinal injury, and more inflammation[58]. In addition, VDR-/- mice had an increased bacterial burden and mortality, more easily detectable levels of IL-6 and elevated NF-κB activity in intestinal epithelia after *Salmonella* infection[59]. For IL-10-/- mice models, double knockout mice (double IL-10/VDR knockout) developed more severe IBD than single VDR-/- and IL-10-/- mice[60].

In addition, in colitis mice models treated with trinitrobenzene sulfonic acid (TNBS), treatment with the vitamin D analogue calcitriol could down-regulate the pro-inflammatory response[61]. These animal studies proved the importance of vitamin D and VDR in inflammation of the gut and in the pathogenesis of IBD.

Nevertheless, IBD also leads to some clinical symptoms, such as impaired absorption of nutrients, abdominal pain, and dysbacteriosis including *clostridium difficile* infection[62], which can influence the absorption of vitamin D. Although many of the studies described above stated that vitamin D levels influence the development of IBD, we still cannot ignore that vitamin D levels are likely affected by IBD. More prospective studies are needed to better understand causation between vitamin D levels and IBD.

We have summarized the relationship between IBD and vitamin D levels, ultraviolet exposure, and geographical distribution. However, some other factors may also influence the morbidity of IBD. Seasonal variation is one factor that we did not analyze. Kini et al reported that serum 25(OH)D levels were lower in winter than in summer (35.9±17.5 vs. 69.6±19.0 nmol/L, *P*<0.0005) [63], but found no significant difference in the mean CDAI score between the seasons (103.9±76.9 vs. 90.2±84.0, *P* = 0.365). Aratari et al noted that the onset of CD symptoms occurred more frequently during spring and summer[64]. However, a Japanese study showed that the onset of symptoms in UC patients frequently occurred during the winter[65]. In addition, smoking has also been proven to be associated with IBD[28]. Ethnicity factors were examined as well. Although vitamin D levels were significantly higher in Caucasians than in non-Caucasians (Asian and Black individuals), no significant association was found between the morbidity and severity of IBD and ethnicity[33]. VDR plays a key role in IBD, and genetic variants of VDR have been shown to be associated with an increased risk of IBD. A meta-analysis showed a significant increase in CD risk for Europeans carrying the TaqI tt genotype and a significant decrease in CD risk for all carriers of the Apal "a" allele[66]. In Asians, the ff genotype of FokI was associated with an increased UC risk[66]. These results indicate that other environmental factors and genetic variation also will affect vitamin D levels and determine the development of IBD.

To our knowledge, this is the first meta-analysis and systemic review to investigate the association of vitamin D levels with IBD as well as the activity of IBD. We reviewed many reports in order to add strength to our study. However, several limitations of our study still exist. First, in most included studies, the diagnostic criteria for IBD were not mentioned, which could directly cause selection bias in the included participants. Pathological diagnosis is considered the gold standard. However, in some studies, IBD was diagnosed under colonoscopy directly, by fecal lactoferrin, by cellular immunology, or another method, and this inconsistency greatly increases the false positive rate and influences the results of included studies. Therefore, in the future, the diagnostic criteria need to be unified. Second, the cut-off level for defining vitamin D deficiency varied among the included studies. The Institute of Medicine recommended 50 nmol/L as the cut-off level[67], and different division standards will directly cause heterogeneity among the results. Third, numerous original studies did not adjust for potentially relevant confounders, such as smoking, latitude, life style, gene polymorphisms, and so on. Any of these factors could lead to bias in the results. In addition, the statistical heterogeneity that appeared in our analysis would also have a small effect on the reliability of our results.

## Conclusions

We have demonstrated that vitamin D levels are lower in IBD patients, suggesting that vitamin D plays an important role in the pathogenesis of IBD. However, we still do not know which specific mechanism plays the main role in this relationship. Potential mechanisms included immune-mediated mechanisms, the anti-inflammatory action of vitamin D, and gene regulation related to vitamin D levels.

## Supporting Information

S1 File PRISMA 2009 Checklist. (DOC)
